# Type of treatment, symptoms and patient satisfaction play an important role in primary care contact during prostate cancer follow-up: results from the population-based PROFILES registry

**DOI:** 10.1186/s12875-021-01567-w

**Published:** 2021-11-04

**Authors:** Barbara M. Wollersheim, Mies van Eenbergen, Kristel M. van Asselt, Laurent M. C. L. Fossion, Evert L. Koldewijn, Jorg R. Oddens, Eric H. Oomens, Bart P. Wijsman, Lonneke V. van de Poll-Franse, Nicole P. M. Ezendam

**Affiliations:** 1 Division of Psychosocial Research and Epidemiology, The Netherlands Cancer Institute, Antoni van Leeuwenhoek Hospital, Amsterdam, The Netherlands; 2 Department of Research, Netherlands Comprehensive Cancer Organization (IKNL), Utrecht, The Netherlands; 3 Department of General Practice, Amsterdam UMC, Location AMC, University of Amsterdam, Amsterdam, The Netherlands; 4 Department of Urology, CHU Brugmann Hospital, Brussels, Belgium; 5 Department of Urology, Catharina Hospital, Eindhoven, The Netherlands; 6 Department of Urology, Amsterdam UMC, location AMC, University of Amsterdam, Amsterdam, The Netherlands; 7 Department of Urology, Amphia Hospital, Breda, The Netherlands; 8 Department of Urology, Elisabeth-TweeSteden Hospital, Tilburg, The Netherlands; 9 Department of Medical and Clinical Psychology, CoRPS – Center of Research on Psychology in Somatic Diseases, Tilburg University, Tilburg, The Netherlands

**Keywords:** Prostate cancer survivors, General practitioner, Primary healthcare contact, Follow-up

## Abstract

**Background:**

With the increasing attention for the role of General Practitioners (GPs) after cancer treatment, it is important to better understand the involvement of GPs following prostate cancer treatment. This study investigates factors associated with GP contact during follow-up of prostate cancer survivors, such as patient, treatment and symptom variables, and satisfaction with, trust in, and appraised knowledge of GPs.

**Methods:**

Of 787 prostate cancer survivors diagnosed between 2007 and 2013, and selected from the Netherlands Cancer Registry, 557 (71%) responded to the invitation to complete a questionnaire. Multivariable logistic regression analyses were performed to investigate which variables were associated with GP contact during follow- up.

**Results:**

In total, 200 (42%) prostate cancer survivors had contact with their GP during follow-up, and 76 (16%) survivors preferred more contact. Survivors who had an intermediate versus low educational level (OR = 2.0) were more likely to have had contact with their GP during follow-up. Survivors treated with surgery (OR = 2.8) or hormonal therapy (OR = 3.5) were also more likely to seek follow-up care from their GP compared to survivors who were treated with active surveillance. Patient reported bowel symptoms (OR = 1.4), hormonal symptoms (OR = 1.4), use of incontinence aids (OR = 1.6), and being satisfied with their GP (OR = 9.5) were also significantly associated with GP contact during follow-up.

**Conclusions:**

Education, treatment, symptoms and patient satisfaction were associated with GP contact during prostate cancer follow-up. These findings highlight the potential for adverse side-effects to be managed in primary care. In light of future changes in cancer care, evaluating prostate cancer follow-up in primary care remains important.

## Background

The number of prostate cancer survivors is increasing due to early detection, better treatment outcomes, and the ageing of the population [1, 2]. Men who are treated for prostate cancer, may suffer from a range of problems affecting their physical, psychological, and social wellbeing [3, 4]. This growing group of prostate cancer survivors who encounter consequences of the cancer and its treatment imposes a burden on the current healthcare system.

Internationally, health authorities recommend to give General Practitioners (GPs) a more prominent role in the cancer care trajectory of cancer survivors [1, 5, 6]. Many cancer patients are older and they often have one or more chronic condition(s), for which they already consult their GP [4]. Studies have shown an increase in primary healthcare use among patients diagnosed with cancer compared to patients without a history of cancer [7–12]. Especially prostate cancer patients show an increase in primary healthcare use 2 to 5 years after their diagnosis in comparison to matched controls [9]. With increasing attention for the role of GPs post-treatment [6], it is important to better understand the current involvement of GPs following treatment of prostate cancer [13, 14].

Until now, most studies have focused on the number of primary healthcare contacts, the number of drug prescriptions, and some determinants for GP contact like age, clinical characteristics (i.e. type of treatment and tumor stage), number of chronic diseases and general comorbidities [7–12]. Unfortunately, these studies only included breast- colorectal- or mix groups of cancer patients. Consequently, there is no information about prostate cancer specific problems and GP contact post-treatment. We hypothesize that prostate cancer survivors experiencing more severe symptoms, will more often contact their GP during follow-up.

It is often argued that GPs are generalists with a more holistic approach to the problems prostate cancer survivors may encounter post-treatment [2]. National health councils of the United States and several Western countries have proposed to give GPs a greater role in the follow-up of cancer survivors [1, 5, 6, 15]. As more countries and hospitals move towards GP-based cancer survivorship care, it is important to understand the current involvement of GPs after cancer treatment. Previous studies have shown that some cancer survivors indicate barriers to contact their GP after treatment, such as having less confidence/faith in GPs for a timely referral, perceived lack of expertise related to cancer, and lack of proper accessible information about their disease [16, 17]. Studies conducted to date mostly asked patients for their preference for a follow-up care provider and did not investigate patients’ perspective towards their GP with regard to satisfaction, trust, and appraised knowledge about their cancer (treatment) [7–12]. We believe that prostate cancer survivors who positively evaluate their GP (i.e. satisfaction with, trust in, appraised knowledge) may already have more contact with their GP after cancer treatment. Such information is crucial to optimize the continuity and coordination of care for prostate cancer survivors.

The specific aims of the study are to (1) describe the number of prostate cancer survivors who have contact with their GP during follow-up, (2) investigate which type of treatment, symptoms or patient factors are associated with GP contact during follow-up, and (3) describe how prostate cancer survivors evaluate their GP with regard to satisfaction with their GP in the phase after treatment, trust in their GP in general and for referrals to the hospital, and knowledge appraisal of their GP about cancer-specific problems and their cancer treatment.

## Methods

### Study design

For this cross-sectional study, we used data from the Patient Reported Outcomes Following Initial treatment and Long-term Evaluation of Survivorship (PROFILES) registry [18]. Patient reported outcomes were collected in PROFILES within a sampling frame of the Netherlands Cancer Registry (NCR) and were linked with clinical data of all individuals newly diagnosed with cancer in the Netherlands.

### Data collection

A detailed description of the data collection has been presented previously [18]. In brief, cancer survivors were informed about the study via a letter from their (previously) attending specialist. Invited participants were given the option of completing either an online or paper questionnaire. This study was part of a broader guideline development and implementation project where data collection took place in 2014 and 2015. Approval was obtained from all study participants, by returning the informed consent form and questionnaire. The procedures were in accordance with the ethical standards of the responsible committee on human experimentation and with the Helsinki Declaration of 1975. Data from the PROFILES registry are freely available for non-commercial scientific research, subject to study question, privacy and confidentiality restrictions, and registration (http://www.profilesregistry.nl).

### Patient sample

We included survivors diagnosed with prostate cancer between September 2007 and April 2013. The start of observation period was at least 6 months after diagnosis. Patients who completed the questionnaire were included in a specialist-based follow-up care program at the hospital, in line with the current Dutch prostate cancer surveillance guideline. Besides, it is important to note that in the Netherlands, the GP is the first contact point for getting healthcare and the gatekeeper to secondary care. Participants were included if they had stage 1–4 prostate cancer and excluded when they were diagnosed with prostate cancer during surgery for bladder cancer as these survivors may not always have been aware of prostate cancer. Survivors had to be between 18 and 85 years at time of survey and being able to read the Dutch language.

### Measures (see Table 1)

**Table 1:** Description of study measures

	Description of items and scales	Scoring, and interpretation of scores:
**Patient and clinical characteristics**	**NCR:** age at time of questionnaire, primary treatment, time since diagnosis, and tumor stage (TNM classification). **Patient reported:** education level, marital status, comorbidity using the self-administered comorbidity questionnaire [19].	Educational level: low (no education and (lower) primary education), intermediate (secondary (vocational) education), and high (higher (vocational) education and university)
**Involvement of GP**		
GP contact	Assessed with one question: ‘Did you have contact with your GP in the period you were recovering from your cancer treatment?’	Yes/no/I don’t know
Preferring more GP contact	Assessed with one question: ‘Would you like to have had (more) contact with your GP during the follow-up phase?’	Yes/no
**Symptoms**	Patient reported, using the EORTC QLQ PR-25 [20]: urinary symptoms, incontinence aid, bowel symptoms, hormonal symptoms, sexual activity, sexual functioning.	0-100: higher scores implies more symptoms or worse functioning
**Patients’ evaluation of their GP**		
Satisfaction with GP	**Item:** Assessed using a self-developed question: ‘Are (were) you satisfied with your GP in the phase after treatment?’. **Scale:** The item was linearly transformed into a scale.	**Item:** Five-point response format ranging from ‘very satisfied’ to ‘very unsatisfied’. **Scale:** 0-100: higher scores implied more satisfaction with GP
Trust in GP	**Items:** Assessed using two self-developed statements: ‘I have a lot of trust in my GP’ and ‘I trust my GP in referring me to the hospital, when necessary’. **Scale:** The items were combined into one scale (Cronbach’s α = 0.84).	**Items:** Five-point response format ranging from ‘totally agree’ to ‘totally disagree’. **Scale:** 0-100: higher scores implied more trust in GP
Appraised knowledge of GP	**Items:** Assessed using three self-developed statements: ‘I think my GP has sufficient knowledge of the side effects and consequences of the cancer treatments’, ‘I think my GP has sufficient knowledge to decide whether it is necessary to refer me to the hospital for my complaints’, and ‘I think my GP knows which medical specialists are experts in assisting people with cancer’. **Scale:** The items were combined into one scale (Cronbach’s α = 0.75).	**Items:** Five-point response format ranging from ‘totally agree’ to ‘totally disagree’. **Scale:** 0-100: higher scores implied higher knowledge rating of GP

Abbreviations. *NCR* Netherlands Cancer Registry, *NA* Not Applicable, *GP* General Practitioner, *EORTC QLQ PR-25* European Organization for Research and Treatment of Cancer Quality of Life Questionnaire – Prostate Cancer Module

#### Patient and clinical characteristics

Sociodemographic and clinical variables were obtained from the NCR: age at time of questionnaire, primary treatment, time since diagnosis, and tumor stage (TNM classification). Education level, marital status, and comorbidity at time of the survey [19] were obtained from the questionnaires.

#### Involvement of GP

The involvement of GPs during the follow-up phase of prostate cancer survivors was assessed with two questions. The first question assessed GPs contact: ‘Did you have contact with your GP in the period you were recovering from your cancer treatment?’. The second question assessed whether prostate cancer survivors preferred more contact with their GP: ‘Would you like to have had (more) contact with your GP during the follow-up phase?’

#### Symptoms

Symptoms were assessed using the prostate cancer specific module of the European Organization for Research and Treatment of Cancer core (EORTC QLQ PR-25) [20].

#### Patients’ evaluation of their GP

Patients’ evaluation of their GP consisted of three items: satisfaction with, trust in, and appraised knowledge of GP. The items were derived from self-developed questions and pilot-tested on patients. The items were linearly transformed into scales, whereby a higher score implied more satisfaction with, more trust in or higher knowledge rating of their GP.

### Statistical analysis

Descriptive statistics were used to describe the demographics, clinical characteristics, symptoms, and patients’ evaluation of their GP. Chi-square and t-tests were used to test the differences between prostate cancer survivors according to having been in contact with their GP during follow-up. Interaction terms were assessed between patients’ satisfaction with their GP and symptoms on contact with the GP during follow-up. In this manner, we were able to evaluate whether symptoms influences the association between patients’ satisfaction with their GP and contact with the GP during follow-up (dependent variable). Subgroup analysis were done to test the differences between survivors according to having preferred more contact with their GP during follow-up.

Multivariable logistic regression analyses were performed to investigate the independent association between contact with the GP during follow-up (yes/no), as the dependent variable, and the independent variables that were determined a priori: patient and treatment characteristics (i.e. age, educational level, marital status, number of comorbid conditions, primary treatment, and time since diagnosis), symptoms (i.e. urinary, bowel and/or hormonal symptoms, sexual activity, incontinence aid, and sexual functioning), and patients’ evaluation of their GP (i.e. satisfaction with GP, trust in GP, and appraised knowledge of GP). Variables were included into the model in three blocks: first (block A), patients and treatment characteristics were added to the model; secondly (block B) symptoms were added to the model, and thirdly (block C) the variables comprising the patient’ evaluation of GPs were added to the model. We chose to include variables into the model in separate steps to better understand the *additional* effects of the variable sets in block B and C. In order to better interpret the parameter estimates of the symptoms scales, we used 10-point odds ratio increase. A 10-point effect measure for the symptom scales would make it easier to understand whether there are any clinically relevant effect estimates in the symptoms scales. Further, block B and C were analyzed without the conditional items ‘incontinence aid’ and ‘sexual functioning’, as that would severely limit the number of patients included in the models. In block B and C we performed a sub-analysis for patients who completed the question regarding incontinence aid (*N* = 139) and the questions regarding sexual functioning (*N* = 223).

SPSS V.15.0 was used for the statistical analyses. A *p* ≤ 0.05 was considered statistically significant and 95% CIs were reported when appropriate. Missing data were handled by pairwise deletion. We have listed the missing data in the footnote of the tables and figures.

## Results

Of the 787 prostate cancer survivors eligible for the study, 557 (71%) survivors responded to the questionnaire. For the current analysis, we included all 475 patients who had complete information on GP contact during follow-up. More details about the representativeness of the study sample were published elsewhere [21].

In total, 200 (42%) prostate cancer survivors had contact with their GP during the follow-up phase, and 76 (16%) prostate cancer survivors preferred more contact with their GP during this phase (Table 2).

**Table 2:** Demographics and clinical characteristics according to having had contact with a general practitioner during follow-up

	Contact with GP during follow-up		
	Yes: *n* = 200 (42%)	No: *n* = 275 (58%)	***p***-value
**Demographics**			
Age at time of questionnaire^a^, M (SD)	71.2 (7.6)	71.3 (7.4)	0.78
Education level^a^			0.76
Low	66 (34.0)	99 (36.7)	
Intermediate	76 (39.2)	97 (35.9)	
High	52 (26.8)	74 (27.4)	
Marital Status^a^			0.07
Partner	179 (90.9)	233 (85.3)	
No partner	18 (9.1)	40 (14.7)	
Number of comorbidities^a^			0.84
No comorbidities	57 (29.1)	83 (30.4)	
1 comorbidity	73 (37.2)	105 (38.5)	
≥ 2 comorbidities	66 (33.7)	85 (31.1)	
Preferring more contact with GP during follow-up^a^	27 (35.5)	49 (64.5)	0.20
**Clinical characteristics**			
Primary treatment^a^			< 0.001
Surgery	80 (40.0)	73 (26.5)	
Radiotherapy	25 (12.5)	41 (14.9)	
Hormonal therapy	29 (14.5)	21 (7.6)	
Radiotherapy and hormonal therapy	31 (15.5)	48 (17.5)	
Active surveillance	27 (13.5)	63 (22.9)	
Watchful waiting	4 (2.0)	5 (1.8)	
Other/unknown	4 (2.0)	24 (8.7)	
Time since diagnosis in years^a^, M (range)	4.4 (1.8–7.8)	4.3 (1.8–8.1)	0.39
Tumor stage^a^			0.003
I	31 (16.2)	79 (31.2)	
II	91 (47.6)	105 (41.5)	
III	44 (23.0)	47 (18.6)	
IV	25 (13.1)	22 (8.7)	

Notes: Percentages for a given variable do not sum up to 100% if the variable contained missing data

^a^Missing data: Age at time of questionnaire = 0; educational level = 11; marital status = 5; number of comorbidities = 6; preferring more contact with the GP during follow-up = 12; primary treatment = 0; time since diagnosis = 0; tumor stage = 31

Abbreviations: *GP* General Practitioner, *M* mean, *SD* standard deviation

**Fig. 1:**
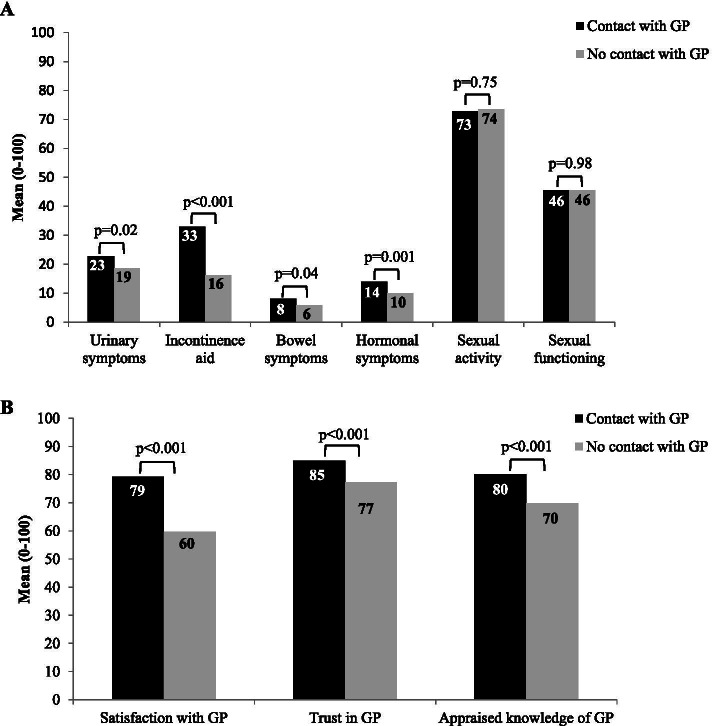
Differences between survivors who had contact and survivors who had no contact with their general practitioner during follow-up. Note: Subgroup analyses (t-tests) were used to test the differences between prostate cancer survivors who had contact with their GP during follow-up and prostate cancer survivors who did not had contact with their GP during follow-up on (**A**) urinary symptoms, incontinence aid, bowel symptoms, hormonal symptoms, sexual activity and sexual functioning, and (**B**) patients’ satisfaction with their GP, patients’ trust in their GP, and the appraised knowledge of GPs according to patients. Missing data: urinary symptoms = 28; (conditional item) incontinence aid = 328; bowel symptoms = 33; hormonal symptoms = 28; sexual activity = 45; (conditional item) sexual functioning = 243; satisfaction with GP = 12; trust in GP = 31; appraised knowledge of GP = 87. Abbreviations: GP = General Practitioner

Prostate cancer survivors who had contact with their GP during follow-up, more often reported urinary symptoms (mean 23 versus 19; *p* = 0.02), bowel symptoms (mean 8 versus 6; *p* = 0.04), hormonal symptoms (mean 14 versus 10; *p* = 0.001), and more often made use of incontinence aids (mean 33 versus 16; *p* < 0.001) than survivors without GP contact during follow-up (Fig. 1A). Prostate cancer survivors who had contact with their GP during follow-up were also more satisfied with their GP (mean 79 versus 60; *p* < 0.001), had more trust in their GP (mean 85 versus 77; *p* < 0.001), and appraised the knowledge of their GP higher (mean 80 versus 70; *p* < 0.001) than those without GP contact during follow-up (Fig. 1B). There was no significant interaction between satisfaction with GP and symptoms on contact with the GP during follow-up.

Subgroup analysis among prostate cancer survivors who preferred to have had more contact with their GP during follow-up showed that these survivors reported more urinary problems, bowel problems, hormonal problems and problems regarding their sexual functioning than those who did not prefer to have had more contact with their GP during follow-up (data not shown).

Multivariable analyses showed that prostate cancer survivors who had an intermediate educational level were more likely to have had contact with their GP during follow-up than survivors who had a low educational level (OR = 2.0; *p* < 0.05) (Table 3). In addition, prostate cancer survivors who were treated with surgery (OR = 2.8; *p* < 0.01) or hormonal therapy (OR = 3.5; *p* < 0.05) were more likely to had contact with their GP during follow-up compared to survivors who were treated with active surveillance. Besides, survivors who experienced bowel symptoms (OR = 1.4; *p* < 0.05), hormonal symptoms (OR = 1.4; *p* < 0.01) or incontinence (OR = 1.6; *p* = 0.001) were more likely to have contact with their GP during follow-up compared to those without these symptoms. Finally, prostate cancer survivors who indicated to be satisfied with their GP were more likely to had contact with their GP during follow-up compared to survivors who indicated not to be satisfied with their GP (OR = 9.5; *p* < 0.001).

**Table 3:** Multivariable regression analyses of factors associated with general practitioner contact during follow-up among prostate cancer survivors (*N* = 475)

	Multivariable association		
	Block A (*n* = 458)	Block B (*n* = 409)	Block C (*n* = 409)
	OR (95% CI)	OR (95% CI)	OR (95% CI)
**Patient and treatment characteristics**			
Age at time of questionnaire	1.0 (0.9:1.0)	1.0 (0.9:1.1)	1.0 (0.9:1.1)
Education level			
Low	1 (ref)	1 (ref)	1 (ref)
Intermediate	1.3 (0.8:2.0)	1.3 (0.8:2.2)	2.0 (1.1:3.4)*
High	1.1 (0.7:1.8)	1.1 (0.7:1.9)	1.7 (0.9:3.1)
Marital Status			
No partner	1 (ref)	1 (ref)	1 (ref)
Partner	1.5 (0.8:2.8)	1.3 (0.7:2.6)	2.0 (0.9:4.2)
Number of comorbidities			
No comorbidities	1 (ref)	1 (ref)	1 (ref)
1 comorbidity	1.0 (0.6:1.6)	1.0 (0.6:1.6)	0.9 (0.5:1.6)
≥ 2 comorbidities	1.2 (0.7:1.9)	1.0 (0.5:1.7)	0.9 (0.5:1.8)
Primary treatment			
Active surveillance	1 (ref)	1 (ref)	1 (ref)
Surgery	2.7 (1.5:4.9)***	2.9 (1.5:5.5)***	2.8 (1.4:6.0)**
Radiotherapy	1.6 (0.8:3.3)	1.7 (0.8:3.7)	1.6 (0.7:3.7)
Hormonal therapy	3.1 (1.4:6.8)**	3.1 (1.3:7.4)**	3.5 (1.3:9.5)*
Radiotherapy and hormonal therapy	1.7 (0.9:3.2)	1.6 (0.7:3.4)	1.6 (0.7:3.8)
Watchful waiting	1.8 (0.5:7.6)	2.8 (0.6:12.9)	2.7 (0.5:14.2)
Other/unknown	0.4 (0.1:1.3)	0.3 (0.1:1.3)	0.2 (0.1:1.0)
Time since diagnosis in years	1.0 (0.9:1.2)	1.1 (0.9:1.2)	1.1 (0.9:1.2)
**Symptoms (per 10 units)**			
Urinary symptoms	–	1.0 (0.9:1.2)	0.9 (0.8:1.1)
Bowel symptoms	–	1.2 (1.0:1.5)	1.4 (1.1:1.9)*
Hormonal symptoms	–	1.3 (1.0:1.6)*	1.4 (1.1:1.9)**
Sexual activity	–	0.9 (0.9:1.0)	0.9 (0.8:1.0)
Incontinence aid^a^ (*n* = 139)	–	1.3 (1.1:1.6)**	1.6 (1.2:2.1)***
Sexual functioning^a^ (*n* = 223)	–	1.0 (0.9:1.2)	1.1 (0.9:1.2)
**Patients’ evaluation of their GP**			
Satisfaction with GP	–	–	9.5 (5.2:17.2)***
Trust in GP	–	–	1.3 (0.4:3.9)
Knowledge of GP	–	–	1.4 (0.5:4.6)

Note: reference category for symptoms = having no symptoms or being not active/functioning; reference category for patients’ evaluation of their GP = being not satisfied with GP, having no trust in GP, and low appraised knowledge of GP

^a^Block B and C were analyzed without the conditional items concerning ‘incontinence aid’ and ‘sexual functioning’. Sub-analyses were performed for patients who completed the question regarding incontinence aid and the questions regarding sexual functioning. The effects of the variables in the sub-analyses were corresponding with the regular analyses (for some variables the significance changed slightly, but this was probably due to under power)

Abbreviations: *OR* Odds Ratio, *CI* Confidence interval, *GP* General Practitioner, *ref* reference category, -- not applicable; *p ≤ 0.05; **p ≤ 0.01; ***p ≤ 0.001

## Discussion

The current study showed that 42% of the prostate cancer survivors had contact with their GP during oncologic follow-up. Furthermore, 16% of the survivors preferred more contact with their GP during this phase. In line with our hypothesis, GP contact in prostate cancer survivors was mostly related to type of treatment (surgery and hormonal therapy), prostate cancer-specific problems, and higher satisfaction with ones GP.

Prostate cancer survivors who have been treated with surgery or hormonal therapy compared to patients under active surveillance seem to seek more often follow-up care from their GP. Remarkably, survivors consulted their GP more often independently from prostate cancer treatment-specific problems. For patients treated with hormonal therapy, this can be explained by the fact that GPs in the Netherlands are often involved in the administration of hormonal therapy. Interestingly, survivors treated with radiotherapy seek less contact with their GP during follow-up. Patients treated with radiotherapy have frequent contact with their radiotherapists during and after treatment. This may decrease the need for contact with the GP. Nevertheless, GPs still seem to play a role in the follow-up care of prostate cancer survivors treated with surgery or hormonal therapy. Since the GP is the first contact-point for (cancer) patients [2], they should be more involved in the assessment of cancer-related problems in collaboration with secondary care.

Not surprisingly, this study demonstrates that prostate cancer treatment-specific problems, like bowel symptoms, hormonal symptoms and urinary incontinence are associated with GP contact post-treatment. These findings are in line with previous studies among breast- and colorectal cancer patients that showed that reasons to contact a GP was associated with treatment-related side effects [8, 11]. It is important that treatment-related side effects are managed in order to maintain higher quality of life, preserve relationship and social activities, and prevent or reduce potential anxiety and depression [3]. Our findings highlight the potential for these adverse side-effects to be investigated and, if possible, managed in primary care.

We hypothesized that the number of comorbidities would also be associated with GP contact. In our study we did not find this association, even though studies have shown that prostate cancer patients more often have a chronic condition, which is often managed by their GP [4, 22]. Nevertheless, Heins and colleagues also found no association between GP contact and the presence of a chronic condition [9]. Perhaps comorbidities have less influence in this group with a relatively high age and a high prevalence of comorbidities.

Unexpectedly, we did not find any association between sexual activity and sexual functioning and GP contact. Sexual dysfunction is one of the most disappointing and distressing outcomes following prostate cancer treatment [23, 24]. We hypothesized that survivors would seek follow-up care from their GP when experiencing sexual problems [21]. Apparently, prostate cancer survivors with sexual problems do not seek care for their sexual problems or they contact their oncologist at the hospital. Subgroup analysis showed that survivors who had problems with sexual functioning preferred to have had more contact with their GP during follow-up. Even though this was based on a sub-sample, this suggests that survivors feel a barrier to speak to their GP about sexual problems. This may be because survivors were too embarrassed to raise psychosexual concerns [25], or they do not know that they could go to their GP with these complaints. Future research should offer more insight into the needs of prostate cancer survivors in managing sexual problems throughout follow-up. Clear guidelines or training on how survivors and healthcare providers should manage sexual problems could help guide men and their partners. Nurse specialists could also play a role in the communication between primary- and secondary care. In recent years, nurse specialists have played a more prominent role in post-treatment cancer care. It is therefore valuable if future studies investigate the role of nurse specialists.

Moreover, the potential for GPs to play a more prominent role in the follow-up may be related to the survivor’s appreciation of their GP. Our study implies that satisfaction with ones GP was associated with seeking follow-up care with the GP. Future studies should further investigate the effect of satisfaction and possible inequalities between prostate cancer survivors.

This study has several limitations to note. First, the patients’ evaluation of their GP was assessed using self-developed questions. Unfortunately, there were no existing questionnaires that included items about patient’s satisfaction with their GP. Second, the timeframe of the dependent variable occurred prior to the independent variable *symptoms*. Symptoms were measured over the past week, while GP contact was measured as follow-up care after treatment. However, as symptoms are generally rather stable after 12 months post-treatment, we believe our conclusions are still valid. Third, selection bias may have occurred as a result of non-participation which could influence the generalizability of the results. Finally, due to the cross-sectional design of the study, we have to interpret the observed associations with caution. For example, the question remains whether survivors went to their GP because they were satisfied with their GP or were they satisfied with their GP because they went to their GP more often.

Strengths of the current study include a large population-based study sample with a high response rate. To our knowledge, this is the first study demonstrating an association between prostate cancer specific problems and GP contact post-treatment.

## Conclusions

In conclusion, this study shows that prostate cancer survivors who had an intermediate educational level, who were treated with surgery or hormonal therapy and those who reported prostate cancer-specific symptoms (i.e. bowel symptoms, hormonal symptoms and urinary incontinence) have more contact with their GP after treatment. Given the satisfaction of cancer survivors with their GP, this study suggests that GPs can have a more formal role in the follow-up of prostate cancer survivors. However, it must be clear to prostate cancer survivors that they can consult their GP when dealing with cancer-specific symptoms. Also GPs should be more equipped with support and training for post-cancer treatment care. Especially for managing sexual problems, GPs may be well placed in assisting men and their partners in engaging in interventions to address sexual problems. In the light of future changes in cancer care, evaluating follow-up care among prostate cancer survivors remains important.

## Data Availability

The dataset used and analyzed during the current study will be available from the corresponding author (stored in a data repository at the Netherlands Cancer Institute) on reasonable request.

## Abbreviations

GP: General Practitioner
PROFILES: Patient Reported Outcomes Following Initial treatment and Long-term Evaluation of Survivorship
NCR: Netherlands Cancer Registry
